# Effects of Citrus Fruit Juices and Their Bioactive Components on Inflammation and Immunity: A Narrative Review

**DOI:** 10.3389/fimmu.2021.712608

**Published:** 2021-06-24

**Authors:** Elizabeth A. Miles, Philip C. Calder

**Affiliations:** ^1^ School of Human Development and Health, Faculty of Medicine, University of Southampton, Southampton, United Kingdom; ^2^ National Institute for Health Research (NIHR) Southampton Biomedical Research Centre, University Hospital Southampton National Health Service (NHS) Foundation Trust and University of Southampton, Southampton, United Kingdom

**Keywords:** immunity, inflammation, infection, cytokine, oxidative stress, vitamin C, folate, bioactives

## Abstract

The immune system provides defence to the host against pathogenic organisms. A weak immune system increases susceptibility to infections and allows infections to become more severe. One component of the immune response is inflammation. Where inflammation is excessive or uncontrolled it can damage host tissues and cause pathology. Limitation of oxidative stress is one means of controlling inflammation. Citrus fruit juices are a particularly good source of vitamin C and folate, which both have roles in sustaining the integrity of immunological barriers and in supporting the function of many types of immune cell including phagocytes, natural killer cells, T-cells and B-cells. Vitamin C is an antioxidant and reduces aspects of the inflammatory response. Important bioactive polyphenols in citrus fruit juices include hesperidin, narirutin and naringin. Hesperidin is a glycoside of hesperetin while narirutin and naringin are glycosides of naringenin. Hesperidin, hesperetin, naringenin, naringin and narirutin have all been found to have anti-inflammatory effects in model systems, and human trials of hesperidin report reductions in inflammatory markers. In humans, orange juice was shown to limit the post-prandial inflammation induced by a high fat-high carbohydrate meal. Consuming orange juice daily for a period of weeks has been reported to reduce markers of inflammation, including C-reactive protein, as confirmed through a recent meta-analysis. A newly emerging topic is whether polyphenols from orange juice have direct anti-viral effects. In summary, micronutrients and other bioactives present in citrus fruit juices have established roles in controlling oxidative stress and inflammation and in supporting innate and acquired immune responses. Trials in humans demonstrate that orange juice reduces inflammation; its effects on innate and acquired immunity require further exploration in well-designed trials in appropriate population sub-groups such as older people.

## Introduction – The Importance of Immunity and the Role of Inflammation

**Figure 1:**
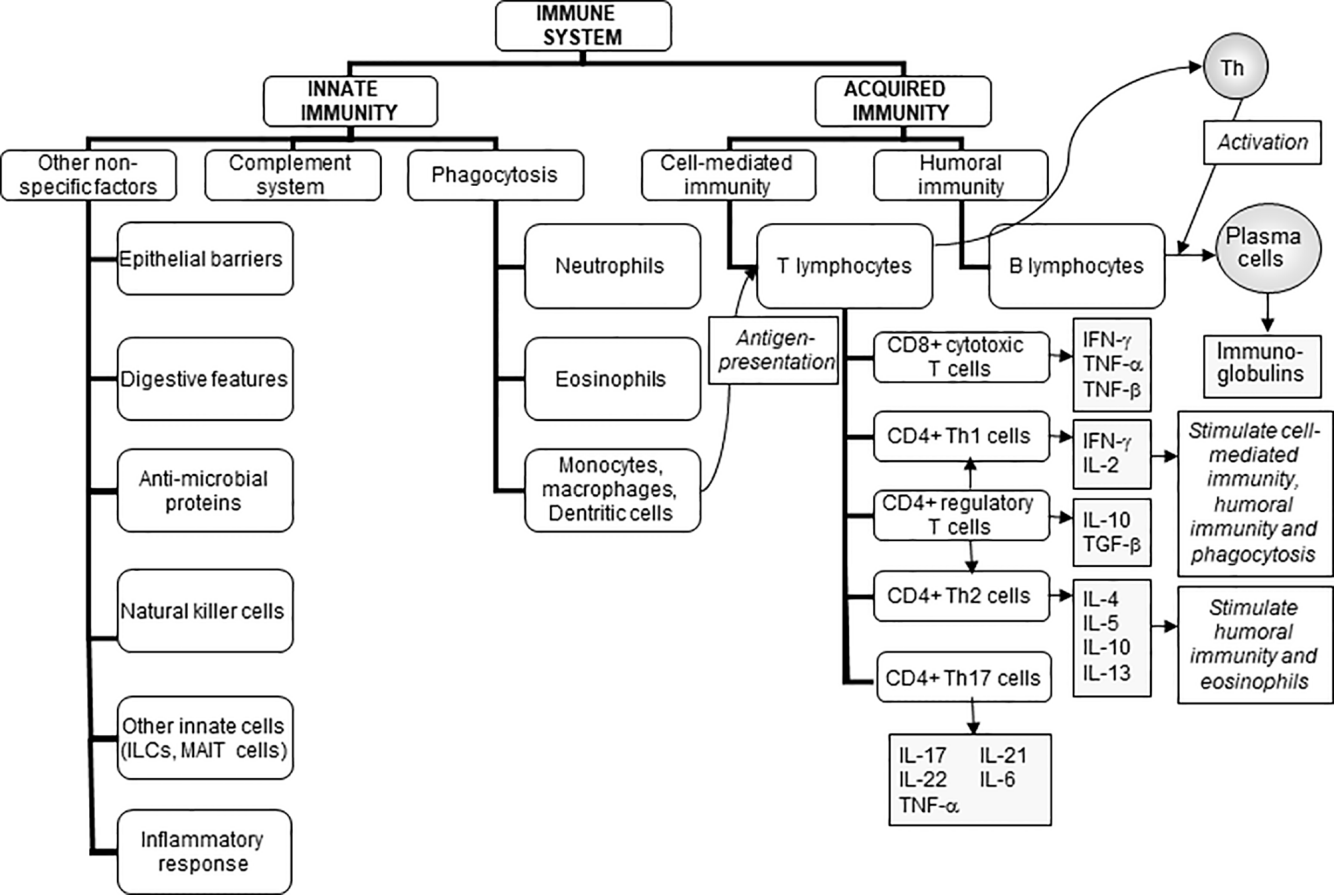
The components of the immune system and their division into innate and acquired immunity. IFN, interferon; IL, interleukin; ILCs, innate lymphoid cells; MAIT, mucosal associated invariant T; TGF, transforming growth factor; TNF, tumour necrosis factor. Taken from (1).

The role of the immune system is to protect the individual against pathogenic organisms including bacteria, viruses, fungi, and parasites. There is a wide array of potentially threatening organisms in the environment. Thus, in order to provide effective protection, the human immune system has evolved to include many different cell types and communicating molecules, and multiple functional responses. The immune system has four general actions. Firstly, it acts as a barrier keeping microbes from entering the body. Examples of barriers include the skin; the mucosal lining of the gastrointestinal, respiratory, and genitourinary tracts; the acid pH of the stomach which kills many bacteria; and anti-microbial proteins in secretions such as tears and saliva. Secondly, the immune system acts to recognise microbes and to identify whether they are harmful or not. Recognition can be of general structural features of microbes (called molecular patterns) or of specific and unique microbial antigens. The mechanism of recognition involves ligand-receptor pairs, but these are different for recognition of molecular patterns compared with recognition of specific antigens and the type of response that is initiated is also different; this is elaborated further in this section. Thirdly, the immune system acts to eliminate those microbes identified as being harmful; this involves the destructive actions of various types of immune cell. Fourthly and finally, the immune response generates immunological memory. This involves long-term maintenance of memory T lymphocytes (T cells) and B lymphocytes (B cells) so that, if there is re-exposure to the harmful microbe, the immune response becomes faster and stronger than it was for the original response. The generation of immunological memory is the basis of vaccination. These complex and sophisticated actions can be achieved because the human immune system is comprised of many cell types (**Figure 1**) (1), each with their own individual functional capabilities. These different cell types interact with one another as part of the immune response to assure effective protection of the host from pathogens. The immune system may be classified in different ways, most commonly into innate (or natural) and acquired (or adaptive) immunity (**Figure 1**).

Innate (sometimes called natural) immunity includes the barrier functions and the cells involved in recognition of molecular patterns on microbes (these are called microbe-associated molecular patterns or MAMPs) and the subsequent destruction of those microbes. Examples of MAMPs include the cell wall lipopolysaccharides of Gram-negative bacteria and the peptidoglycans of Gram-positive bacteria. These general structural features are recognised by pattern recognition receptors; toll-like receptors are examples of pattern recognition receptors but there are many others. The typical response following recognition would be to engulf the microbe carrying the MAMP by the process of phagocytosis, with subsequent destruction of the microbe within lysosomes by the so-called respiratory burst which generates destructive reactive oxygen species. Neutrophils, monocytes, macrophages, and dendritic cells are all phagocytic cells. The inflammatory response is also triggered by this process, with the aim of creating an environment that is hostile to the invading microbes; in fact, an inflammatory response can be triggered by isolated MAMPs, not only by microbes bearing MAMPs. Note too that the inflammatory response can be damaging to the host if it is not properly controlled and many pathologies involve adverse inflammation (2). Components of the engulfed microbes appear on the surface of the phagocytes and are displayed (“presented”) to antigen-specific helper T cells; phagocytes capable of such display are called antigen-presenting cells.

Acquired (sometimes called adaptive) immunity includes antigen recognition and antigen-specific effector functions such as the proliferation of T cells, the killing of virally-infected cells by cytotoxic T cells, and the production of antibodies by B cells. Acquired immunity can be further sub-classified into cell-mediated immunity involving T cells and humoral immunity involving B cells and antibody production. There are multiple types of T cells, each with different roles in the immune response (**Figure 1**).

Innate and acquired immunity are linked. As mentioned already, phagocytic cells such as macrophages and dendritic cells, which are part of innate immunity, act as antigen-presenting cells, whereby they process and then present antigens derived from engulfed microbes to antigen-specific T cells so eliciting acquired immunity. Conversely, cytokines produced by activated T cells regulate the activity of innate immune cells. Furthermore, antibodies produced by B cells coat microbes, making the process of phagocytosis more efficient. Thus, there is bidirectional communication between innate and acquired immunity and this can involve both cell-to-cell contact and production of, and responses to, chemical mediators.

It is obvious that effective defense against pathogenic organisms requires a well-functioning immune system. Consequently, individuals with weakened immune systems are at increased risk of becoming infected and of infections being more serious, even fatal. Seriously immunocompromised individuals must live their lives in protected environments, where they are guarded against exposure to harmful microbes. The immune system also plays a role in assuring immunologic tolerance towards non-threating exposures including harmless microbes (e.g. commensal bacteria in the gastrointestinal tract) and food components. If this tolerance is lost, adverse immune reactions are triggered.

**Figure 2:**
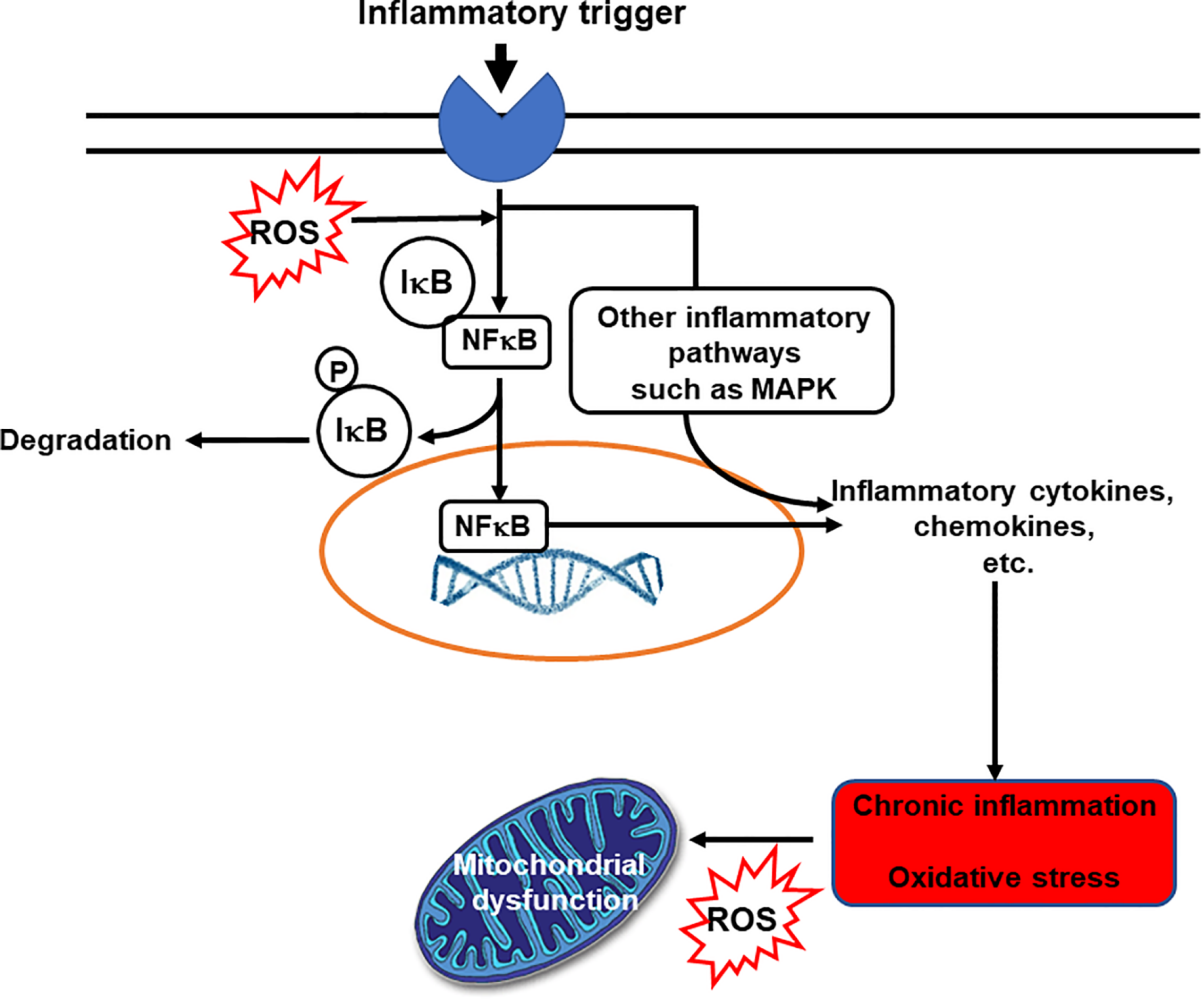
The bidirectional links between inflammation and oxidative stress. Reactive oxygen species (ROS) can act as inflammatory trigger initiating inflammation. On the other hand inflammation induces oxidative stress. IκB, inhibitory subunit of NFκB; MAPK, mitogen-activated protein kinase; NFκB, nuclear factor kappa-light-chain-enhancer of activated B cells; P, phosphate; ROS, reactive oxygen species.

Inflammation is an essential and normal component of the innate immune response. In general, inflammation acts to create an environment that is hostile to pathogens, it initiates pathogen killing, and it causes changes in the metabolism of the host. Many immune cell types play roles in the inflammatory response, which involves the production of, and responses to, a vast number of chemical mediators. The cardinal signs of inflammation are redness, swelling, heat, pain and loss of function. These are caused by the cellular activation and chemical mediator release that occur during the initiation and perpetuation of the inflammatory response. The chemical mediators released from cells during inflammation include lipids (e.g. prostaglandins, leukotrienes, endocannabinoids, platelet activating factor), proteins (e.g. cytokines, chemokines), reactive oxygen species (e.g. superoxide anion, hydrogen peroxide), amino acid derivatives (e.g. histamine, nitric oxide) and enzymes (e.g. matrix proteases) depending upon the cell types present, the nature of the inflammatory stimulus, the anatomical site involved, and the stage during the inflammatory response. Although the inflammatory response is designed to be damaging to pathogens, the cellular activities involved and the chemical mediators produced can cause damage to host tissues. Fortunately, therefore, inflammation is normally self-limiting and typically resolves rapidly. This is because various inhibitory mechanisms are activated as inflammation runs its course. Loss of the regulatory processes involved in resolution of inflammation can result in excessive, inappropriate or on-going inflammation that can cause irreparable damage to host tissues leading to pathology and disease (3). Inflammation is an important component of a wide array of human conditions including classic chronic inflammatory diseases like rheumatoid arthritis, inflammatory bowel diseases, allergy and asthma which are all controlled or treated with varying degrees of success with anti-inflammatory medications (2, 3). Inflammation is also involved in cardiovascular diseases, metabolic diseases, neurodegenerative disorders and cognitive decline; in many cancers; and in ageing (4–6). The relationship between inflammation and oxidative stress is bidirectional: oxidative stress induces inflammation and inflammation induces oxidative stress (**Figure 2**). Hence, agents that act to reduce oxidative stress can also be anti-inflammatory.

**Figure 3:**
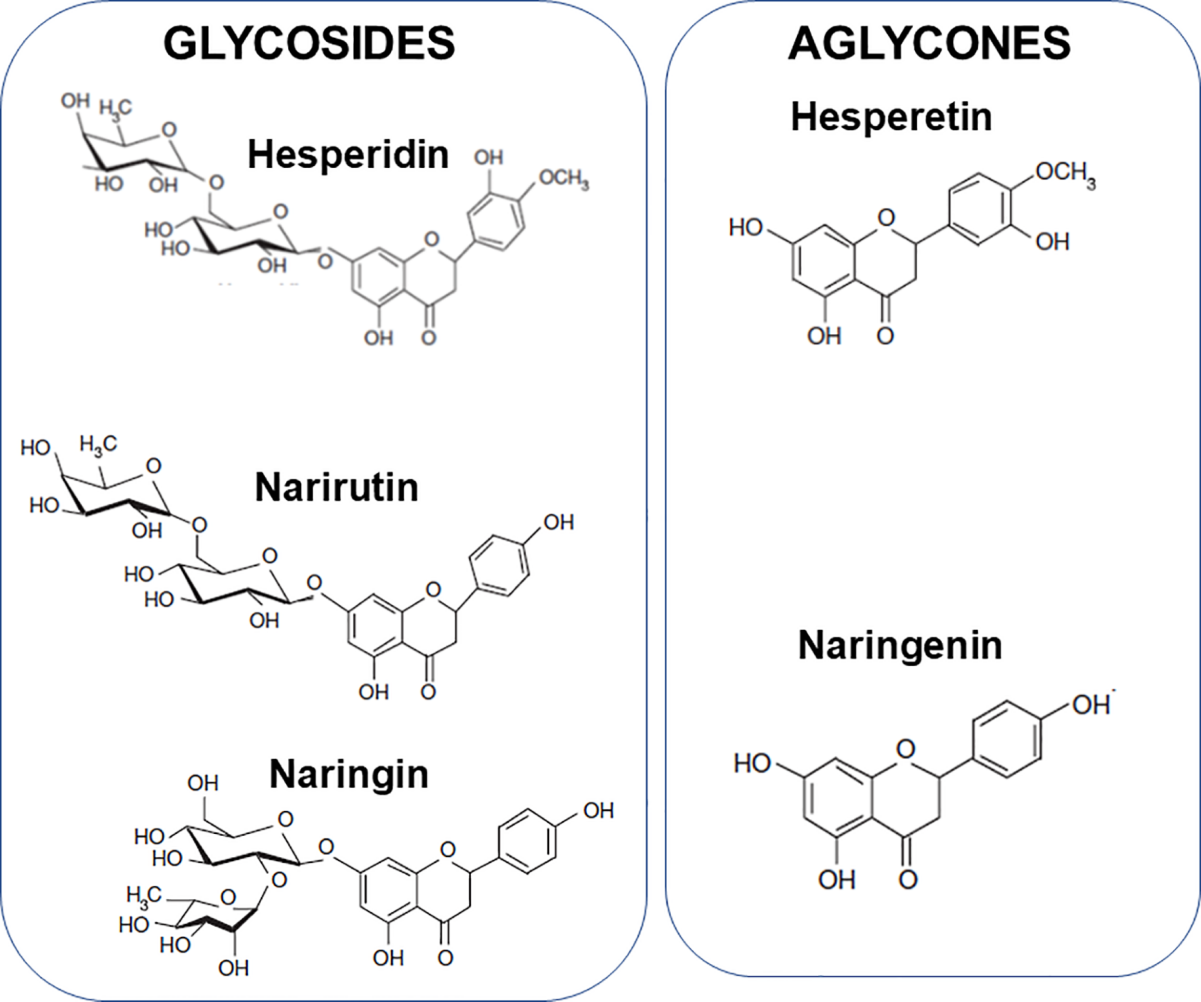
Structure of hesperidin, narirutin and naringin and the aglycones hesperetin and naringenin.

The aim of this article is to review the literature that relates to the modulation of components of the immune response, including inflammation, by citrus fruit juices and their bioactive components and to describe the mechanisms involved. The bioactive components considered are vitamin C, folate, hesperidin, narirutin and naringin. Hesperidin is a glycoside of hesperetin (**Figure 3**) and is present in high amounts in sweet oranges, lemons, limes, and tangerines; it comprises 90% of the flavanones in orange juice. Narirutin and naringin are glycosides of naringenin (**Figure 3**). Naringin is the major flavonoid in grapefruits with far lower amounts seen in sweet oranges, lemons, limes and tangelos. Narirutin is found in grapefruits and in sweet oranges, tangerines and tangelos.

## Biomarkers of Immunity

Clearly the immune system is highly complex involving many different cell types and subtypes and functional responses, and the production of, and responses to, many chemical mediators (**Figure 1**). Each of these components can be measured experimentally. In humans this is most often performed using blood samples, although some immune biomarkers can also be measured in other accessible fluids including saliva. It is important to note that most immunologic activity does not take place in the bloodstream but in lymphoid organs such as the spleen and lymph nodes, or in tissues such as the gut mucosa and lungs. As a consequence of this, only the minority of immune cells are in the bloodstream at any one time. It is generally agreed that there is no single marker of either the status or the functional capacity of the immune system (7–10). In most human settings, circulating cell numbers, their activation state, and responses to an *ex vivo* challenge can be, and are frequently, measured. There are normal ranges for circulating immune cell numbers and immunoglobulin concentrations, but there are no normal ranges for immune cell functional responses. Assessments of the functional capacity of the immune system can be made by:

- measuring specific cell functions *ex vivo* (i.e., of cells isolated and studied in short- or long-term culture);
- measuring *in vivo* responses to challenge, for example by measuring the changes in the concentrations of antibodies in the bloodstream (or saliva) in response to an *in vivo* immunologic challenge such as vaccination;
- measuring the incidence, duration, and severity of infections.

Expert groups have summarised and evaluated a large number of immune function assays commonly used as markers in human intervention studies (9, 10). Markers classified as being of high suitability were vaccine-specific serum antibody concentrations, the delayed-type hypersensitivity response, vaccine-specific or total secretory immunoglobulin (Ig) A in saliva, and the response to attenuated pathogens. Markers classified as being of medium suitability included natural killer cell cytotoxicity, oxidative burst of phagocytes, lymphocyte proliferation, and the cytokine pattern produced by activated immune cells. Other markers were classified as being of low suitability. Albers et al. (9) stated that “since no single marker allows conclusions to be drawn about the modulation of the whole immune system, except for the clinical outcome of infection itself, combining markers with high and medium suitability is currently the best approach to measure immunomodulation in human nutrition intervention studies”. With regard to inflammation, the total number of leukocytes (white blood cells) and circulating C-reactive protein (CRP) concentration are regarded as valid measures and may be supported by measuring concentrations of pro- and anti-inflammatory cytokines, chemokines and lipid mediators (2, 4–6). CRP at low concentrations requires measurement kits with high sensitivity to be used. As such, CRP measured with these kits is sometimes referred to as high-sensitivity CRP; it is important to note that this does not indicate a different type of CRP but merely indicates the nature of the assay used to measure CRP. Patterns and clusters of such markers may be more robust biomarkers of inflammatory state and inflammatory response than individual markers or small numbers of markers. In addition, markers of cellular activation and gene expression profiles can be used to gain information about entire pathways of immune activation or inflammatory state and can also provide insights into mechanisms involved in any immune/inflammatory challenge or in modulation of the response to such challenges.

## Nutrients Within Citrus Fruit Juices That Are of Particular Relevance to the Immune System

Citrus fruit juices contain a wide range of micronutrients (vitamins and minerals) and bioactive compounds; a comprehensive nutrient composition of orange juice has been provided elsewhere (11). Several of these micronutrients are important in immune function support (12–16) and citrus fruit juice is a particularly good source of two of these, vitamin C and folate. The European Food Safety Authority permits claims of “contributes to the normal function of the immune system” for both vitamin C and folate (17). The vitamin C content of orange juice is quoted as 31 mg/100 g and 40 mg/100 g juice stored at ambient or chilled temperature, respectively (11), but is known to vary by season and fruit variety. Chanson-Rolle et al. (18) present a compositional analysis of a number of commercial and home-made orange juices; all home-made juices were produced in Spain using Valencia oranges. They found that commercial orange juice contained about 15% less vitamin C than home-made orange juice (40.5 ± 10.1 mg/100 ml vs 47.8 ± 8.59 mg/100 ml). This difference may relate to variations in production and storage. De Rycker et al. (19) report on a survey of the vitamin C content of 615 samples of orange juice sourced globally: average content was 45 ± 9.8 mg/100 ml with a range of 12.0 to 72.1 mg/100 ml. Ashchoff et al. (20) report a vitamin C content of freshly squeezed orange juice of 49.4 mg/100 g with > 98% of this present as ascorbic acid. While storage of orange juice (whether fresh or commercially-squeezed) results in a decline in vitamin C content, less so if oxygen exposure is limited and temperature is reduced (21), industry standards require that at least 200 mg/litre of vitamin C must be present at the end of shelf life [European Fruit Juice Association, personal communication].

The total folate content of orange juice is quoted as 32 μg/100 g and 22 μg/100 g juice stored at ambient or chilled temperature, respectively (11). Chanson-Rolle et al. (18) report that commercial orange juice contained about 27% less folate than home-made orange juice (25 ± 5.8 μg/100 ml vs 34 ± 5.8 μg/100 ml, respectively). Others report similar values for folate [e.g. 16 to 30 μg/100 g (22)] in orange juice. The main form of folate in orange juice is 5-methyl-tetrahydrofolate (22), which is stable over normal shelf life (22).

In addition to micronutrients, citrus fruit juices contain a number of polyphenolic flavonoid compounds of relevance to the immune system. The concentration of total polyphenols was found to be similar between commercial and home-made orange juices (63.3 ± 5.85 mg/100 ml vs 62.9 ± 5.94 mg/100 mL, respectively) (18). Hesperidin is the main polyphenol in orange juice. De Rycker et al. (19) report on a survey of the hesperidin content of 231 samples of orange juice sourced globally: average content was 52 ± 17.5 mg/100 ml with a range of 10.9 to 116.0 mg/100 ml. Ashchoff et al. (20) report hesperidin and narirutin contents of fresh orange juice as 29.3 and 5.3 mg/100 g, respectively. Bestwick et al. (23) and Gattuso et al. (24) report data for multiple phytochemicals in orange juices, while Grosso et al. (25) report such data for blood orange juice, which contains a different profile of polyphenolic compounds compared with regular orange juice. Li et al. (26) report that the hesperidin and narirutin contents of a commercial blood orange juice were 80.2 ± 2.7 and 9.5 ± 0.1 mg/100 ml, respectively. Blood orange juice contained 2.4 ± 0.13 mg/100 ml anthocyanins (26). Phenolic compounds in orange juice have been noted to decline minimally during optimal low temperature storage (21).

## Bioavailability of Bioactives From Citrus Fruit Juices

The bioavailability of bioactives from food and beverages is important if they are to exert a physiological effect or a health benefit, although they may also act *via* effects on gastrointestinal microbiota. The bioavailability of vitamin C (comprising ascorbic acid and dehydroascorbic acid) ranges between 80% and 100% at normal intakes (27). Folate bioavailability is discussed in detail elsewhere (28); bioavailability varies depending upon the exact chemical form and the food matrix but can be high. There is some evidence that a maximum of 30% of an ingested dose of hesperidin might be absorbed in the small intestine (29, 30). The majority of an ingested dose of hesperidin or narirutin is believed to reach the colon, where they are hydrolysed by the colonic microbiota, primarily yielding their corresponding aglycones hesperetin and naringenin, which are then absorbed by colonocytes. After conjugation with glucuronic acid or sulphate, they are released into the bloodstream (31, 32). Reported urinary flavanone recoveries are poor: only 4.1–5.4% and 2.1–12.5% of total hesperidin and narirutin intake has been estimated to be bioavailable (31, 33–35). However, a substantial portion of the flavanone aglycones is further metabolized to similarly bioavailable catabolites by the colonic microbiota (36). Thus, the total bioavailability once all chemical forms are considered may be higher and has even been stated to be almost 100% of the ingested dose (37). Nevertheless, a considerable interindividual variability in the absorption and metabolism of citrus flavanones has been noted, most likely associated with difference in gut microbiota (38). The availability of hesperidin from orange juice appears to be greater than for whole oranges (38), while levels of hesperidin are three times greater in commercially-squeezed orange juice compared with home-squeezed which results in higher blood levels of hesperitin (39).

## Evidence for Effects of Citrus Fruit Juices and Their Major Bioactives on Inflammation and Immunity

### Introductory Comments

Fruits, fruit extracts and fruit juices are good sources of micronutrients and of bioactive phytochemicals. Many of these play roles in supporting the immune response, in controlling inflammation and in preventing or controlling oxidative stress which promotes inflammation and harms the immune response. In a randomised controlled trial, older people (65 to 85 years of age) who consumed 5 or more portions of fruits and vegetables per day had a better response to the vaccine against pneumococcus than those consuming 2 or less portions per day (40). Bub et al. (41) compared the effects of two blends of fruit juice on immune parameters in healthy men; the juices used were blends of apple, orange, mango and berry juice or of apple, orange, mango, lime and apricot juice along with green tea. Both were matched for total polyphenol content although the nature of the polyphenols differed. Intervention duration was two weeks. Both juices increased lymphocyte proliferation, interleukin (IL)-2 production and natural killer cell activity compared with baseline. The effects of a dried encapsulated fruit and vegetable extract on immune function have been tested in several studies. After 80 days, this extract increased lymphocyte proliferation and natural killer cell activity in older men and increased IL-2 production in those who smoked cigarettes (42). A 77-day randomised controlled trial in university students reported that the extract increased γδ T cells in the blood stream and resulted in fewer symptoms of the common cold (43). γδ T cells are a distinct sub-population of T cells that are relatively uncommon but are most abundant in the gut mucosa where they contribute to the intraepithelial lymphocyte population. They are considered to be regulatory cells that link innate and adaptive immunity. A randomised controlled trial over 28 weeks in middle-aged men reported that the encapsulated extract improved markers of oxidative stress and decreased the inflammatory marker CRP (44); there was also a tendency to less illness in those consuming the extract compared with the control group. Finally, a large randomised controlled trial (n = 543) over 8 months in healthcare staff aged 18 to 65 years reported a reduction in days of symptoms of the common cold in those consuming the extract compared with the control group (45). Taken together these studies indicate that fruits and vegetables, their juices and concentrates of their juices, can beneficially modify immune responses, inflammation, and oxidative stress in humans.

### Orange Juices and Inflammation

#### Postprandial Studies

It is well described that the post-prandial period can be accompanied by an elevation in the blood concentrations of markers of inflammation including various cytokines and adhesion molecules (46). This post-prandial inflammation is exaggerated by meals high in sugar, total fat or saturated fat and is believed to enhance cardiovascular risk (46). The effects of including a specific component (e.g. orange juice) in a test meal can be tested (“acute” effect) or the effects of chronic consumption (weeks, months) of a specific component on the response to a standard test meal can be investigated (“chronic” effect). Acute effects of orange juice consumption on inflammatory markers have been evaluated in postprandial studies. In the study by Ghanim et al. (47), orange juice was compared with energy-matched drinks containing glucose or fructose or a saccharin-containing control. Glucose promoted an increase in reactive oxygen species production by neutrophils and in activation of the pro-inflammatory transcription factor nuclear factor kappa-light-chain-enhancer of activated B cells (NFκB) in mononuclear cells. However, these effects were not seen with fructose, orange juice or saccharin. Plasma CRP declined one hour after consuming orange juice. These observations indicate that orange juice itself does not induce an acute inflammation.

Further research by this group (48) considered the effect of orange juice on the post-prandial inflammatory response induced by a high fat-high carbohydrate meal. Adding orange juice to a standard meal reduced the post-prandial generation of reactive oxygen species by neutrophils compared with the meal plus water or the meal plus glucose. Orange juice totally mitigated the post-prandial rise in p38 mitogen-activated protein kinase (MAPK), phosphorylated p38 MAPK (the active form of MAPK) and p47phox (a subunit of NADPH oxidase responsible for reactive oxygen species production) in mononuclear cells, all molecular markers of enhanced inflammation, as well as the elevation in matrix metalloproteinase (MMP)-9 mRNA in mononuclear cells. Plasma MMP-9 concentration was not elevated with orange juice unlike in the other two groups, while the post-prandial elevation in toll-like receptor (TLR) 2 and TLR4 mRNA and protein in mononuclear cells seen with glucose did not occur with orange juice. Endotoxemia occurred in the meal plus water and the meal plus glucose groups by not in the meal plus orange juice group. These observations suggest that orange juice mitigates the acute pro-inflammatory effects of a high fat-high carbohydrate meal. A comparison of test meals accompanied by water, cream, glucose or orange juice confirmed the protective effects of orange juice (49): unlike the meals with cream or glucose, the meal with orange juice did not elevate tumour necrosis factor (TNF)-α or IL-1β mRNA or NFκB activation in mononuclear cells. Furthermore, unlike the meal with cream, the meal with orange juice did not elevate TLR4 mRNA or protein in mononuclear cells. Taken together, these findings suggest that inclusion of orange juice with a meal could minimize postprandial oxidative stress and inflammation.

#### Intervention Studies

The influence of chronic intervention with orange juice on inflammatory markers has been studied. In an uncontrolled study in 12 young adults, Sánchez-Moreno et al. (50) found that drinking two glasses of orange juice (500 ml) a day for 14 days reduced the plasma concentrations of prostaglandin E_2_ and 8-epi-prostaglandin F_2α_ and tended to reduce the concentration of CRP. In healthy overweight men, the consumption of 500 ml orange juice daily for 4 weeks did not affect serum concentrations of several inflammatory markers (CRP, IL-6, soluble intercellular adhesion molecule (ICAM)-1, soluble vascular cell adhesion molecule (sVCAM)-1) (51), although blood pressure was lowered and vascular function improved. The orange juice intervention modulated the expression of 3,422 genes many of which are involved in chemotaxis, adhesion, and cell infiltration (52). Buscemi et al. (53) found reduced plasma concentrations of CRP, IL-6, and TNF-α in non-diabetic individuals with increased cardiovascular risk after one week of daily consumption of 500 ml blood orange juice. Endothelial function, which was measured as flow-mediated dilation, significantly improved in these subjects. Asgary et al. (54) compared effects of fresh and commercial orange juice in a cross-over study in 22 healthy adults who consumed 500 ml of orange juice twice daily for 4 weeks: serum concentrations of CRP, sVCAM-1 and sE-selectin, but not IL-6, were decreased by both types of juice with no difference between them. In another study, 750 ml orange juice daily for 8 weeks lowered circulating CRP and raised IL-12, but did not affect IL-4, IL-10, TNF-α or interferon (IFN)-γ, in both normal weight and overweight adults (55). A second study with the same design (750 ml red fleshed orange juice daily for 8 weeks) also reported a reduction in CRP concentration in both normal weight and overweight individuals (56). Patients with hepatitis C who consumed 500 ml of orange juice daily for 8 weeks showed a reduction in plasma CRP concentration, although the starting value was higher than in the control group (57). Both normal polyphenol containing orange juice (299 mg polyphenols/L) and high polyphenol orange juice (745 mg polyphenols/L) provided at 500 ml daily for 12 weeks altered plasma lipid mediators in healthy participants with greater effects with the high polyphenol orange juice (58). A recent meta-analysis of the effects of orange juice on risk factors for cardiovascular disease reported that orange juice significantly decreased CRP levels (7 trials; weighted mean difference: -0.467 mg/L, 95% confidence interval: -0.815, -0.120, p = 0.008) compared to placebo (59). However, there was significant between-study heterogeneity: subgroup analysis identified that studies conducted on individuals with metabolic disease, that used more than 500 ml orange juice/day and that had an intervention duration less than 8 weeks showed a greater reduction in CRP levels (59) The greater effect of the higher intake of orange juice makes sense because higher intakes will provide greater amounts of the bioactive components. The greater effect of shorter (than 8 week) durations is perhaps counterintuitive but may be explained by loss of compliance in longer duration studies.

### Orange Juices and Immunity

Whilst a number of studies have investigated the effect of orange juices on inflammation, there are almost no studies of the effects on markers of innate or acquired immunity beyond inflammation. Perche et al. (60) conducted a trial in 24 healthy men of mean age 56 years who underwent 3 x 4 week treatment periods separated by 3 week washout periods. The three treatments were 500 ml orange juice daily, 500 ml isocaloric control drink daily or 500 ml of the control drink plus 292 mg hesperidin in capsules daily. There was no effect on blood immune cell phenotypes, the percentage of T cells and B cells activated with an immune stimulant *ex vivo, ex vivo* production of IL-2 and IL-4 by stimulated leukocytes, natural killer cell activity, or reactive oxygen species production by stimulated neutrophils. It is important to note that this study was conducted in healthy men and that it may be difficult to show improvements in immune function in healthy individuals.

### Vitamin C, Inflammation and Immunity

#### Overview

Vitamin C is an essential nutrient that acts primarily as a water-soluble antioxidant. It is a cofactor for a number of enzymes including the lysyl and prolyl hydroxylases required for stabilization of the tertiary structure of collagen. Hence, vitamin C is vital for maintaining epithelial integrity. Severe vitamin C deficiency results in scurvy, which is potentially fatal. Scurvy is characterized by weakening of collagenous structures, resulting in poor wound healing, and impaired immunity; individuals with scurvy are highly susceptible to potentially fatal infections such as pneumonia (61). Cells of the immune system actively accumulate vitamin C against a concentration gradient, resulting in cellular concentrations that can be up to 50- or 100-times those seen in plasma (62–64). For example, neutrophils can accumulate vitamin C to achieve intracellular concentrations of 1 mM or more (62, 65). This suggests that vitamin C is of some importance to immune cells. Vitamin C has anti-inflammatory effects, in part because of its role as an antioxidant, and also has roles in several aspects of immunity, including leucocyte migration to sites of infection, phagocytosis and bacterial killing, natural killer cell activity, T lymphocyte function and antibody production. There are a number of comprehensive reviews of the role of vitamin C in immunity and host susceptibility to infection (66, 67).

#### Vitamin C and Barrier Function

Vitamin C is actively accumulated into epidermal and dermal cells *via* sodium-dependent vitamin C transporters, suggesting that it has important functions within the skin. The effects of scurvy demonstrate the key role of vitamin C in maintaining barrier integrity. Vitamin C promotes collagen gene expression in fibroblasts (68–72) and promotes fibroblast proliferation and migration which is essential for tissue remodelling and wound healing (73, 74). Vitamin C intervention studies in humans have shown enhanced vitamin C uptake into skin cells (75, 76) and enhanced oxidant scavenging activity of the skin (76, 77). The elevated antioxidant status of the skin following vitamin C supplementation could potentially protect against oxidative stress induced by UV irradiation and environmental pollutants (78, 79).

#### Vitamin C and Inflammation

Although cells of the immune system contain high concentrations of vitamin C, these can be decreased upon cellular stimulation, resulting in a loss of antioxidant protective mechanisms. An altered balance between oxidant generation and antioxidant defences can lead to changes in multiple signalling pathways, with the pro-inflammatory transcription factor NFκB playing a central role (**Figure 2**). Oxidants can activate NFκB leading to continued synthesis of oxidative species and other inflammatory mediators (80) (**Figure 2**). Vitamin C can diminish both oxidant generation and NFκB activation (81) and can modulate inflammation through redox-sensitive cell signalling pathways (82, 83) or by directly protecting important structural components of the cell from damage (84). In accordance with these proposed anti-inflammatory actions, vitamin C can modulate production of inflammatory cytokines. For example, it decreased lipopolysaccharide-induced production of TNF-α and IFN-γ, and increased anti-inflammatory IL-10 production, by human lymphocytes in culture (85). Vitamin C treatment reduced activation of microglial cells and decreased the synthesis of the pro-inflammatory cytokines TNF-α, IL-6, and IL-1β (86). Addition of vitamin C to peripheral blood monocytes isolated from patients with pneumonia decreased the generation of the pro-inflammatory cytokines TNF-α and IL-6 (87). These findings are all consistent with an anti-inflammatory action of vitamin C. However, providing 1 g/day vitamin C (with and without vitamin E) to healthy volunteers was found to enhance IL-10, IL-1 and TNF-α production by blood mononuclear cells following stimulation with LPS (88, 89).

#### Vitamin C and Cellular Aspects of Innate Immunity

Chemotaxis describes the movement of immune cells into infected tissues which is an early step in innate immunity. Neutrophils express many receptors for different chemo-attractants, enabling them to sense and rapidly respond to signals indicating infection or tissue damage (90). Leukocytes from vitamin C deficient guinea pigs show impaired chemotactic responses (91–94). Studies with large doses of vitamin C in patients with recurrent infections and impaired leukocyte chemotaxis showed restoration of chemotaxis (95–101). Supplementation of healthy volunteers with vitamin C has also been shown to enhance neutrophil chemotactic ability (84, 102–104). For example, in one study, provision of vitamin C through the diet (250 mg/day) increased neutrophil chemotaxis by 20% (104). Furthermore, supplementation of elderly women with 1 g/day vitamin C, in combination with vitamin E, enhanced neutrophil functions, including chemotaxis (105). Phagocytosis is the process of engulfing pathogens which are subsequently destroyed within intracellular vacuoles, in part by the oxidative burst. Neutrophils, monocytes, macrophages, and dendritic cells are all phagocytic cells. Neutrophils from vitamin C deficient guinea pigs have an impaired ability to kill microbes (91, 92, 106), linked to defective phagocytosis and/or respiratory burst (106–108). Dietary vitamin C (250 mg/day) enhanced neutrophil respiratory burst by 20% in human participants with low vitamin C status (104), while the combination of vitamins C and E increased both phagocytosis and respiratory burst of neutrophils in older people (105). Vitamin C maintains or enhances natural killer cell activity (109, 110).

#### Vitamin C and Lymphocyte Functions

Like phagocytes, B and T lymphocytes accumulate vitamin C to high levels *via* specific transporters (111, 112). Jacob et al. (113) showed that a vitamin C-deficient diet in healthy young adult humans decreased mononuclear cell vitamin C content by 50% and decreased the T lymphocyte-mediated immune responses to recall antigens, suggesting a strong causal link between lymphocyte vitamin C content and lymphocyte function. Vitamin C seems to be important in the differentiation and maturation of immature T cells (114, 115), effects which may relate to epigenetic modifications (115–117). *In vitro* studies have indicated that incubation of lymphocytes with vitamin C promotes T lymphocyte proliferation (85, 114) and increases antibody production (118). Treatment of guinea pigs with vitamin C increased T cell proliferation (119) and enhanced antibody levels during immunization (120, 121). One human study reported that vitamin C supplementation (1 g/day for 73 days) increased serum IgM, IgG and IgA levels (122), although that effect was not seen in another study that used 1, 2 and 3 g vitamin C/day (103). However, 1, 3 and 3 g vitamin C/day enhanced *ex vivo* T lymphocyte proliferation (103). Administration of vitamin C to elderly people (500 mg/day for 1 month) was also shown to enhance *ex vivo* T lymphocyte proliferation (123), which was also seen with combinations of vitamin C with vitamins A and/or E (110, 124).

#### Vitamin C and Infection

Vitamin C clearly has benefits in supporting barrier function and both innate and acquired immunity. Furthermore, incubation of virus-infected human and murine fibroblasts with vitamin C enhanced generation of anti-viral IFNs (89, 125–128). A major symptom of scurvy is increased susceptibility to infections, particularly of the respiratory tract, with pneumonia being one of the most frequent complications of scurvy and a major cause of death (61, 66). This suggests that vitamin C likely has a role in protecting against infections, particularly of the respiratory tract. Significant decreases in leukocyte vitamin C levels occur during common cold episodes, with levels returning to normal following the infection (129–132), indicating that vitamin C is utilized during a common cold infection. Administration of high doses of vitamin C (6 g/day) during a common cold episode ameliorated the decline in leukocyte vitamin C, suggesting that administration of vitamin C may be beneficial for the recovery process (129). A meta-analysis of randomised controlled trials (RCTs) identified that vitamin C did not affect incidence of the common cold in the general population (24 RCTs) but decreased incidence in people under heavy short-term physical stress (5 RCTs) (133). Vitamin C shortened the duration of the common cold in all studies (31 RCTs), in adults (13 RCTs) and in children (10 RCTs) and decreased the severity of colds (133). Plasma vitamin C concentrations are reduced in patients with acute respiratory infections, such as pulmonary tuberculosis and pneumonia (134, 135). In elderly people hospitalized because of pneumonia and who were identified to have very low vitamin C levels, administration of vitamin C (200 mg/day for 4 weeks) reduced the respiratory symptom score in the more severe patients (136). In other pneumonia patients, low dose vitamin C (250 to 800 mg/day) reduced hospital stay by 19% compared with no vitamin C supplementation, while higher-dose vitamin C (500 mg to 1.6 g/day) reduced the duration of pneumonia by 36% (137). There was also a positive effect on the chest X-ray, temperature, and erythrocyte sedimentation rate (137). A meta-analysis of 3 RCTs reported a significant reduction in the risk of pneumonia with vitamin C supplementation, particularly in individuals with low dietary intakes (138).

### Folate and Immunity

Folate is essential for the synthesis of RNA and DNA and consequently for cell division, protein synthesis and tissue growth. It is not a surprise therefore that folate is required for the immune system to function. In common with other B vitamins, folate (vitamin B9) is involved in intestinal immune regulation (139, 140), thus contributing to gut barrier function. In fact, folate is essential for the survival of regulatory T cells in the small intestine wall (141), suggesting it plays a role in preventing adverse immune responses at that site. Regulatory T cells express high levels of folate receptor 4 (FR4) and administration of anti-FR4 antibody to mice results in specific reduction in the regulatory T cell population (141), indicating that the folate-FR4 axis is required for regulatory T cell maintenance. *In vitro* culture of regulatory T cells in folate-restricted conditions impaired cell survival, with decreased expression of anti-apoptotic bcl2 molecules, although naïve T cells retained the ability to differentiate into regulatory T cells (142, 143); this suggests that folate is a survival factor for regulatory T cells. Consistent with these findings, dietary deficiency of folate results in reduction of the regulatory T cell population in the small intestine of mice (141, 142). Since regulatory T cells play an important role in the prevention of excessive immune responses (144), mice fed a folate-deficient diet exhibit increased susceptibility to intestinal inflammation (141). Some commensal intestinal bacteria convert folate to 6-formylpterin (145) which may suppress excess mucosal associated invariant T cell responses and prevent excessive allergic and inflammatory responses (146–148).

Folate deficiency in experimental animals also causes systemic immune effects such as thymus and spleen atrophy and lower circulating T lymphocyte numbers: lymphocyte proliferation is also reduced in folate deficiency (149). However, the phagocytic and bactericidal capacity of neutrophils appear unchanged (149). Folate deficiency reduces natural killer cell activity in rats (150) and inhibits the proliferation of human CD8^+^ cytotoxic T lymphocytes *in vitro* (151), effects which would reduce antiviral defences. Folate deficient culture medium resulted in an immature phenotype of murine bone marrow derived dendritic cells that produced less IL-12 and pro-inflammatory cytokines in response to LPS (152). This aberrant maturation of dendritic cells resulted in reduced ability to induce helper T cell responses with low production of cytokines including IL-2, IFN-γ and IL-10 (152). Folate deficiency in mice resulted in poor dendritic cell and spleen cell responses (cytokine production) and altered T cell phenotypes (152), while folate deficiency in rats or mice impairs antibody production (153, 154). Thus, studies in experimental animals demonstrate that folate is essential for the immune system to function properly. Rather less is known about the influence of variations in folate intake or status in human populations and immune outcomes. Congenital isolated malabsorption of folic acid is associated with impairment of both cellular and humoral immunity, and increased infections (155), while suppressed T cell mediated immunity in patients with megaloblastic anaemia with folate deficiency was reversed by folate treatment (156). Critically ill patients with lower folate status had poorer neutrophil phagocytosis than those with higher folate status (157). Likewise malnourished patients with lower folate status had poorer neutrophil function (phagocytosis, bacterial killing) than those with higher folate status and the impaired phagocytosis was corrected by folic acid supplementation (158). Furthermore, the impairment in phagocytosis could be corrected by adding folic acid to the medium of the cultured neutrophils (158). These studies indicate that having sufficient folate is important for the human immune system to function.

Hara et al. (159) reported that serum folate status positively associated with antibody titres following seasonal influenza vaccination, although this association lost significance when the data were adjusted for age. An intervention with high dose folic acid (1.2 mg per day for 12 weeks) in healthy subjects increased lymphocyte folate by 44% (160). Plasma levels of a number of proteins related to immunity were positively associated with folate status both prior to and following intervention (160). Folic acid supplementation increased plasma concentrations of a number of immune-related proteins, including IgM C chain and complement 3 (160). Folate has been a component of several micronutrient mixtures or nutritional supplements that have been reported to increase some, though not all, immune biomarkers (161–163), including those associated with anti-viral defence (164, 165), and to decrease infections (162, 163), although the effects observed cannot, of course, be ascribed to folate. It is also important to note that some studies of micronutrient mixtures that include folate do not show improvements in immune outcomes (166, 167). Nevertheless, it seems clear from the literature that an adequate folate intake and status is required to support the human immune system.

### Hesperetin, Hesperidin, and Inflammation

Hesperetin is the aglycone of hesperidin (**Figure 3**). The anti-inflammatory effects of hesperetin and hesperidin have been examined in several cell culture studies [reviewed by Chanet et al. (168)]. Hesperetin decreased production of TNF-α by lipopolysaccharide-stimulated macrophages in a concentration dependent manner (169, 170); IL-6 production was not affected (170). Hesperitin did not affect expression of the inhibitory subunit of NFκB or inducible nitric oxide synthase in these cells following lipopolysaccharide stimulation and only modestly affected nitric oxide production (169). Increased adhesion of monocytes to endothelial cells and expression of vascular cell adhesion molecule-1 in response to TNF-α treatment were reduced by pretreatment with hesperetin (171). Both hesperetin and hesperidin deceased expression of the adhesion molecule VCAM-1 in TNF-stimulated endothelial cells (172, 173) and decreased monocyte adhesion to endothelial cells (171, 173). Hesperidin also reduced ICAM-1 expression on endothelial cells cultured in high glucose concentrations (174), an effect associated with reduced phosphorylation of the p38 MAPK. Hesperitin decreased IL-1β-induced MMP-3 and IL-6 production by cultured human synovial cells, which was linked to reduced activation of c-Jun N-terminal kinase (175).

Feeding hesperidin to mice for 6 weeks prior to undergoing irradiation resulted in lower concentrations of serum IL-1β, IL-6, and TNF-α compared to the control irradiated group (176). Furthermore, splenocyte proliferation on day 10 after irradiation was enhanced by supplementation with hesperidin and the percentages of CD4^+^ and CD8^+^ lymphocytes tended to increase compared with the normal group (176). This study suggests that hesperidin may enhance immunocompetence and decrease irradiation-induced inflammation in mice.

In a placebo controlled human trial with a crossover design conducted in 24 men and women aged 21 to 65 years with metabolic syndrome, hesperidin (500 mg daily for 3 weeks resulted in significantly lowered plasma concentrations of CRP, serum amyloid A and sE-selectin (171). A controlled trial in 64 patients with type-2 diabetes found that 500 mg/day hesperidin for 6 weeks decreased both CRP and IL-6 concentrations from baseline values (177). In another human study, 292 mg hesperidin daily for 4 weeks modified the gene expression profile of white blood cells (52); hesperidin intake modulated the expression of 1,819 genes many of which are involved in chemotaxis, adhesion, and cell infiltration. In this study over 50% of the genes modulated by orange juice consumption were also modulated by hesperidin, suggesting that hesperidin makes an important contribution to the anti-inflammatory effects of orange juice.

### Naringenin, Naringin, Narirutin, and Inflammation

Naringenin is the aglycone of naringin and narirutin (**Figure 3**). The anti-inflammatory effects of naringenin have been examined in several cell culture and animal feeding studies [reviewed by Chanet et al. (168)]. In cell culture experiments, naringenin has been shown to decrease expression of inducible nitric oxide synthase and cyclooxygenase-2 and to decrease production of TNF-α, IL-1β, IL-6 and prostaglandin E_2_ by lipopolysaccharide-stimulated macrophages (178, 179). Naringenin also reduced expression of inducible nitric oxide synthase and cyclooxygenase-2 and decreased production of prostaglandin E2 and expression of mRNA for TNF-α, IL-1β and monocyte chemoattractant peptide 1 by BV2 microglial cells in culture (180). Naringenin also decreased expression of the adhesion molecule VCAM-1 in TNF-stimulated endothelial cells (172) and decreased monocyte adhesion to endothelial cells (181). Such effects appear to relate to deceased activation of the pro-inflammatory transcription factor NFκB (172, 180, 182) and of MAPKs (180). Inclusion of naringenin in the diet of rabbits fed a high cholesterol diet reduced expression of VCAM-1 and monocyte chemoattractant peptide 1 in the aortic arch (183).

Naringin has also been studied *in vitro* and in animal feeding studies. Naringin deceased expression of the VCAM-1 in TNF-stimulated endothelial cells (172). Naringin also reduced ICAM-1 expression on endothelial cells cultured in high glucose concentrations (174), an effect associated with reduced phosphorylation of the p38 MAPK. Inclusion of naringin in the diet of rabbits fed a high cholesterol diet reduced expression of VCAM-1 and MCP-1 in the aortic arch (183) and reduced expression of ICAM-1 on endothelial cells (184). Inclusion of naringin in the diet of mice fed a high cholesterol diet reduced blood levels of sICAM-1 and sE-selectin (181). Dietary naringin lowered serum TNF-α concentration and increased serum adiponectin in mice ref a high fat diet (185). Dietary naringin dose-dependently decreased serum concentrations of TNF-α, IL-6 and CRP and increased adiponectin concentration in diabetic rats fed a high fat diet compared with diabetic control rats (186). In this same study, naringen increased liver and kidney expression of the anti-inflammatory transcription factor peroxisome proliferator activated receptor-γ and of heat shock protein-27 and -72 and decreased liver, kidney, and pancreas expression of NFκB (186).

Narirutin and naringin both decreased nitric oxide production by lipopolysaccharide-stimulated macrophages and decreased CRP release from incubated rat aortic vascular ring (187). These data suggest that naringenin and its glycosides naringin and narirutin may have similar anti-inflammatory effects.

### Direct Anti-Viral Activities of Citrus Fruit Juice Bioactives

Beyond effects supporting immune function and controlling inflammation, bioactives present in citrus fruit juices may have direct anti-viral effects; these have been highlighted in the context of infection with systemic acute respiratory syndrome coronavirus (SARS-CoV)-2 and the disease that this virus causes, coronavirus disease discovered in 2019 (COVID-19). Angiotensin converting enzyme (ACE) 2 is a transmembrane protein which acts as a receptor for spike protein binding of SARS−CoV-2, enabling cellular entry of the virus. Using *in silico* modelling it was identified that hesperidin can bind with ACE2 and in doing so may make the ACE2-SARS-CoV-2 spike protein structure unstable (188–190). Through this action it is proposed that hesperidin could block SARS-CoV-2 from entering host cells and so could prevent the infection. Hesperidin has also been shown to prevent replication of several viruses including the influenza virus acting through activation of immune-supporting MAPK pathways (191) and in mice it prevented the spread of influenza virus (192). Both hesperidin and hesperetin are able to inhibit key proteases involved in coronavirus replication (193, 194). As reviewed by Tutunchi (195) naringenin exerts similar actions suggesting it too could inhibit viral entry into host cells and subsequent viral replication.

## Integration, Summary, and Conclusions

**Figure 4:**
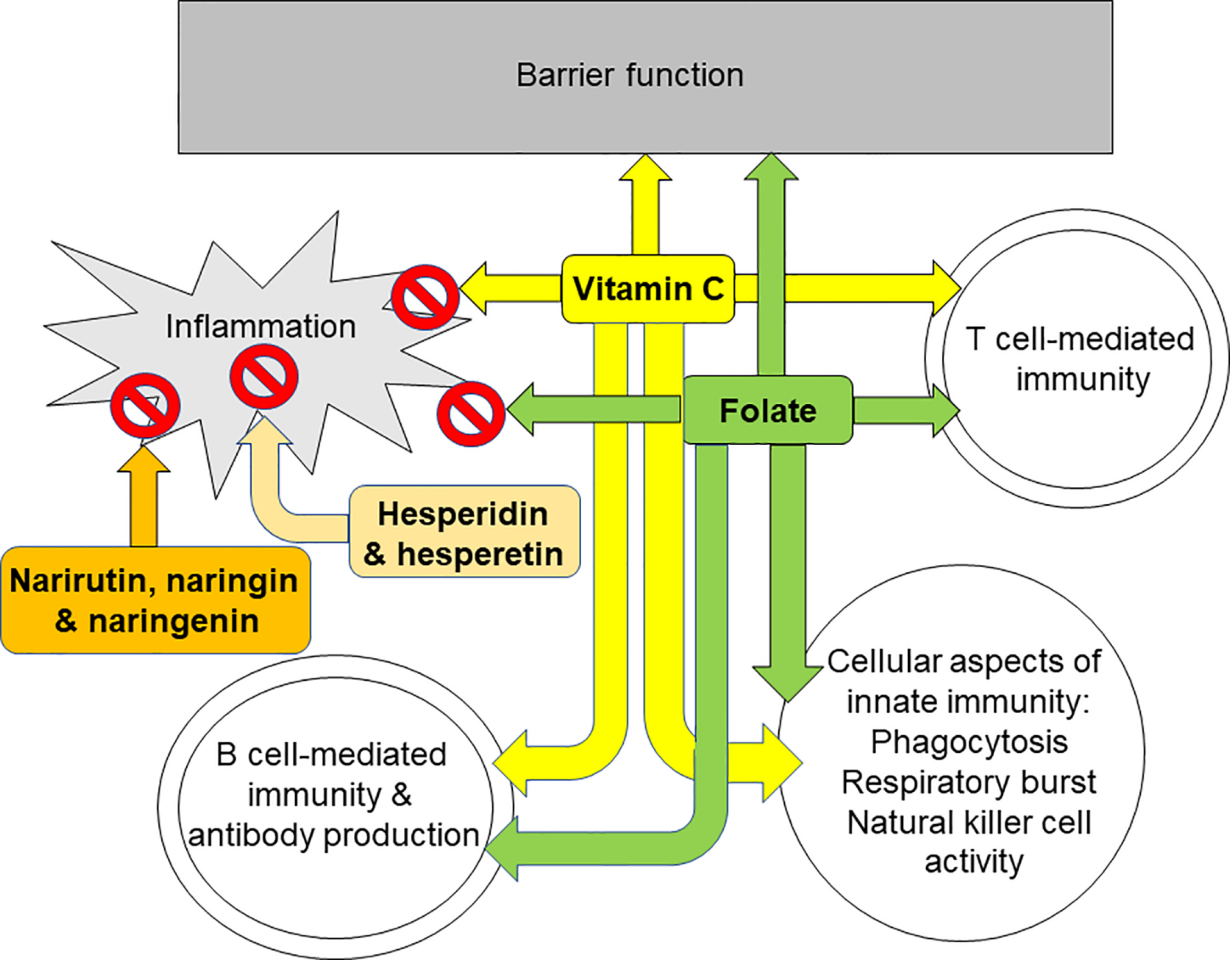
Summary of the effects of orange juice bioactives on different aspects of inflammation and immunity. Vitamin C and folate support barrier function, T cell mediated immunity and B cell mediated immunity. Vitamin C, folate, hesperidin and its aglycone hesperetin, and narirutin and naringin and their aglycone naringenin all reduce inflammation.

The immune system provides defence to the host against pathogenic organisms. It includes barrier functions and capabilities for recognition and elimination of pathogens and for immunologic memory. A weak immune system increases susceptibility to infections and allows infections to become more severe. One component of the immune response is inflammation which is designed to create a hostile environment to pathogens. Generation of oxidative stress is part of the inflammatory response and, in turn, oxidative stress can induce inflammation. Where inflammation is excessive or uncontrolled it can damage host tissues and cause pathology. Hence, an immune response which is appropriate to the challenge and involves controlled inflammation that is self-resolving is optimal. Limitation of oxidative stress is one means of controlling inflammation, hence, antioxidants are often also anti-inflammatory. Nutrition is one of many determinants of the immune response (1, 12–16) including the inflammatory component (4–6). Micronutrients (vitamins and minerals) are especially important for supporting normal immune response (1, 12–16) and plant polyphenols have also emerged as having important roles, not only in helping to control oxidative and inflammatory stress, but also in supporting the activities of the cellular aspects of innate and acquired immunity. Citrus fruit juices contain a wide range of vitamins, minerals, and polyphenols, with 100% orange juices being a particularly good source of vitamin C and folate. Vitamin C and folate both have roles in sustaining the integrity of immunological barriers including the skin and internal mucosal linings (**Figure 4**), while vitamin C is an antioxidant and helps to control inflammation (**Figure 4**). As described earlier, both vitamin C and folate support the function of many types of immune cell including phagocytes, natural killer cells, T-cells, and B-cells (**Figure 4**). In recognising the roles of vitamin C and folate within the immune response, the European Food Safety Authority (and the UK Government post-BREXIT) permit a claim of “contributes to the normal function of the immune system” for both vitamin C and folate (17). To carry this claim, one serving of a food must supply at least 15% of the Nutrient Reference Value of the nutrient, while beverages must supply at least 7.5%. The Nutrient Reference Values for vitamin C and folate are 80 mg and 200 μg respectively. Typical contents of vitamin C and folate in orange juice are 40 to 50 mg/100 ml and 20 to 40 μg/100 ml, respectively, these being influenced by the type of oranges used for making the juice and how the juice is stored. Nevertheless, it is clear that a serving of 100% orange juice would provide sufficient amounts of both vitamin C and folate to carry a permitted immune claim. Important bioactive polyphenols in citrus fruit juices include hesperidin, narirutin and naringin. Hesperidin is a glycoside of hesperetin and narirutin and naringin are glycosides of naringenin (**Figure 3**). Hesperidin, hesperetin, naringenin, naringin and narirutin have all been demonstrated to have anti-inflammatory effects, mainly demonstrated in cell culture and some animal studies; all seem to act, at least in part, through inhibiting activation of the pro-inflammatory transcription factor NFκB. Human trials of hesperidin in people with metabolic syndrome (171) or type-2 diabetes (177) reported reductions in inflammatory markers, including CRP. Hesperidin modified gene expression in white blood cells with significant overlap of the genes modified with those modified by orange juice (52). Thus, citrus fruit juices contain a mix of components that control oxidative stress and inflammation, and support the immune system. In the context of human trials, orange juice has been most widely explored, although specific trials on immunity are scarce. Orange juice was shown to limit the post-prandial inflammation induced by a high fat-high carbohydrate meal (48). Consuming orange juice daily for a period of weeks reduced markers of inflammation, including CRP, as confirmed through a recent meta-analysis (59). One human intervention trial with orange juice failed to find effects on markers of innate or acquired immunity (60); however this trial studied healthy middle aged men and it may be that groups vulnerable to declines in immune function, such as the elderly, may be a better option for this type of trial. Despite the findings of the latter study, in general the effects of orange juice, especially with regard to inflammation, are consistent with those of its component bioactives. A newly emerging topic, driven largely by the SARS-CoV-2 pandemic, is whether polyphenols from orange juice have direct anti-viral effects. There is evidence from *in silico* modelling studies that hesperidin could interfere with SARS-CoV-2 entry into host cells through destabilising the interaction between the virus’ spike protein and ACE2 receptor on host cells (188–190). Furthermore *in vitro* studies identify that hesperidin, hesperetin and naringenin can restrict viral replication acting through inhibition of key enzymes involved in this process (193–195). Whether these effects occur in infected humans at intakes and circulating concentrations of these bioactives consistent with normal fruit juice consumption is uncertain. In this context a clinical trial of hesperidin in people newly infected with SARS-CoV-2 has been registered (196). In summary, micronutrients and other bioactives present in citrus fruit juices have established plausible pathways for controlling oxidative stress and inflammation and for supporting innate and acquired immune responses. Trials in humans demonstrate that orange juice reduces inflammation, while its effects on innate and acquired immunity require further exploration in well-designed trials in appropriate population sub-groups, such as older people.

## Author Contributions

The first draft of the article was prepared by PC. EM provided comment. All authors contributed to the article and approved the submitted version.

## Funding

The University of Southampton received funds from a consortium of orange producers, juice manufacturers and packaging companies based in Europe and Brazil under the umbrella of the European Fruit Juice Association (AIJN). The funders had no influence on the content of the article nor on the decision of where to publish.

## Conflict of Interest

The authors declare that the research was conducted in the absence of any commercial or financial relationships that could be construed as a potential conflict of interest.

## Abbreviations

ACE, angiotensin converting enzyme; COVID-19, coronavirus disease discovered in 2019; CRP, C-reactive protein; FR4, folate receptor 4; ICAM, intercellular adhesion molecule; IFN, interferon; Ig, immunoglobulin; IL, interleukin; MAMP, microbe-associated molecular pattern; MAPK, mitogen-activated protein kinase; MMP, matrix metalloproteinase; NFκB, nuclear factor kappa-light-chain-enhancer of activated B cells; SARS-CoV-2, systemic acute respiratory distress syndrome coronavirus 2; TLR, toll-like receptor; TNF, tumour necrosis factor; VCAM, vascular cell adhesion molecule.
